# Comparative genomic analysis of *Geobacter sulfurreducens* KN400, a strain with enhanced capacity for extracellular electron transfer and electricity production

**DOI:** 10.1186/1471-2164-13-471

**Published:** 2012-09-12

**Authors:** Jessica E Butler, Nelson D Young, Muktak Aklujkar, Derek R Lovley

**Affiliations:** 1 Department of Microbiology, 203 Morrill Science Center IVN, University of Massachusetts, 639 North Pleasant Street, Amherst, MA, 01003, USA

**Keywords:** *Geobacter sulfurreducens* KN400, Comparative genomics, Microbial fuel cell, Extracellular electron transfer

## Abstract

**Background:**

A new strain of *Geobacter sulfurreducens*, strain KN400, produces more electrical current in microbial fuel cells and reduces insoluble Fe(III) oxides much faster than the wildtype strain, PCA. The genome of KN400 was compared to wildtype with the goal of discovering how the network for extracellular electron transfer has changed and how these two strains evolved.

**Results:**

Both genomes were re-annotated, resulting in 14 fewer genes (net) in the PCA genome; 28 fewer (net) in the KN400 genome; and ca. 400 gene start and stop sites moved. 96% of genes in KN400 had clear orthologs with conserved synteny in PCA. Most of the remaining genes were in regions of genomic mobility and were strain-specific or conserved in other *Geobacteraceae*, indicating that the changes occurred post-divergence. There were 27,270 single nucleotide polymorphisms (SNP) between the genomes. There was significant enrichment for SNP locations in non-coding or synonymous amino acid sites, indicating significant selective pressure since the divergence. 25% of orthologs had sequence differences, and this set was enriched in phosphorylation and ATP-dependent enzymes. Substantial sequence differences (at least 12 non-synonymous SNP/kb) were found in 3.6% of the orthologs, and this set was enriched in cytochromes and integral membrane proteins. Genes known to be involved in electron transport, those used in the metabolic cell model, and those that exhibit changes in expression during growth in microbial fuel cells were examined in detail.

**Conclusions:**

The improvement in external electron transfer in the KN400 strain does not appear to be due to novel gene acquisition, but rather to changes in the common metabolic network. The increase in electron transfer rate and yield in KN400 may be due to changes in carbon flux towards oxidation pathways and to changes in ATP metabolism, both of which indicate that the overall energy state of the cell may be different. The electrically conductive pili appear to be unchanged, but cytochrome folding, localization, and redox potentials may all be affected, which would alter the electrical connection between the cell and the substrate.

## Background

The ability to effectively transfer electrons directly onto insoluble, extracellular substances is one of the hallmark characteristics of *Geobacter* species. In the natural environment, *Geobacter* species are commonly the most abundant microorganisms in anaerobic soils and sediments where microbial reduction of insoluble Fe(III) oxides is important [1]. In addition, *Geobacter* species can be used to make microbial fuel cells - devices in which electrical current is harvested from bacteria that grow by transferring electrons from their food source to the anode of the device [1-3]. Pure cultures of *Geobacter sulfurreducens* produce current densities that are among the highest known [4], and *G. sulfurreducens* has been enriched from a number of complex communities growing in biofilms on high-efficiency microbial fuel cells [3]. However, the mechanisms for extracellular electron transfer in *Geobacter* species are poorly understood [1].

Recently, a new strain of *G. sulfurreducens*, designated strain KN400, was isolated that is much more effective than wildtype both in electron transfer to electrodes [5] and in Fe(III) oxide reduction (KA Flanagan, personal communication). Strain KN400 was isolated from a culture that grew by transferring electrons to an anode poised at a very low potential (−200 mV SHE) [5]. Compared to the wildtype strain, when growing in microbial fuel cells KN400 forms biofilms on the anode more rapidly; it produces much higher-density current (7.4 *versus* 1.4 A/m^2^) and power (3.9 *versus* 0.5 W/m^2^) [5]; and it has higher conductivity (350 *versus* 75 μS/cm) [6].

Because it was recently isolated, little is known about how KN400 differs from the wildtype strain. Here we present the analysis of the genome of KN400 [7] and compare it to the genome of wildtype *G. sulfurreducens* PCA [8] in order to provide insight into both the phenotype of enhanced extracellular electron transfer and the evolutionary history of the species. We identify the genes that are unique to each strain and identify polymorphisms between the genomes. We analyze functional enrichment in the set of genes with sequence changes, and focus on differences in genes involved in electron transfer pathways from intracellular oxidation to electron transfer onto extracellular acceptors. We use these analyses to suggest how the metabolic network of the species has evolved and how these differences caused the divergent phenotypes.

## Results and discussion

### Reannotation of the *G. sulfurreducens* strains KN400 and PCA genomes

As published, the *G. sulfurreducens* strain PCA genome was 3.8 Mb with 3466 open reading frames (ORFs) [8] and the *G. sulfurreducens* strain KN400 genome was 3.7 Mb with 3356 ORFs [7]. Because the two genomes were sequenced and annotated several years apart using different methods, we re-annotated both genomes, making several types of corrections in order to assure that comparisons between the two were valid. ORF predictions, start and stop sites of the genes, and functional predictions were compared and reconciled using identical methods on both genomes (Additional file 1: Table S1 and Additional file 1: Table S2).

After the re-annotations, the strain KN400 genome had 3328 ORFs (Additional file 1: Table S1), and the strain PCA genome had 3432 ORFs (Additional file 1: Table S2). These re-annotations were deposited in the National Center for Biotechnology Information genome database under the accession numbers of AE017180.2 for PCA and NC_017454 for KN400. A summary of the basic characteristics of the genomes is given in Table 1.

**Table 1:** **Characteristics of the re-annotated* G. sulfurreducens* genomes**

	**strain KN400**	**strain PCA**
**genome size (bp)**	3714272	3814128
**open reading frames**	3328	3432
**G + C content (%)**	61.3	60.94
**rRNA operons**	2	2
**plasmids**	0	0

### Whole-genome comparison of strain KN400 to strain PCA

Orthologs, proteins predicted to have the same function in both genomes, were identified using a combination of whole-genome alignment and all-versus-all protein sequence alignments. A whole-genome nucleotide alignment of the PCA and KN400 genomes showed that they were syntenic, with no large-scale rearrangements or inversions (Additional file 2: Figure S1). Orthologs were identified for 3180 of the 3328 protein-coding genes in the KN400 genome (Additional file 1: Table S1). A whole-proteome comparison identified orthologs for 3194 genes (Additional file 1: Table S1). The two methods agreed for 3166 (99.6%) of the pairs, with the genome method identifying 14 orthologs with identical sequences: eight transposases, two cytochromes, and a translation elongation factor, as well as three orthologs under 100 amino acids in length (Additional file 1: Table S1).The proteome method identified 28 orthologs with low sequence similarity or alignment over a short region of the sequence (Additional file 1: Table S1). For the analysis of polymorphisms between orthologs presented below, we included orthologs identified by only one method if they aligned over at least 50% of the length of the longer protein, and had a sequence identity of at least 30%.

In total, 3192 of the 3328 ORFs in the KN400 strain genome (96%) have orthologs in the PCA strain genome (Additional file 1: Table S1 and Additional file 1: Table S2). The relative locations of the conserved genes are largely preserved between the genomes (Additional file 2: Figure S1).

### Genes unique to the KN400 strain

**Figure 1:**
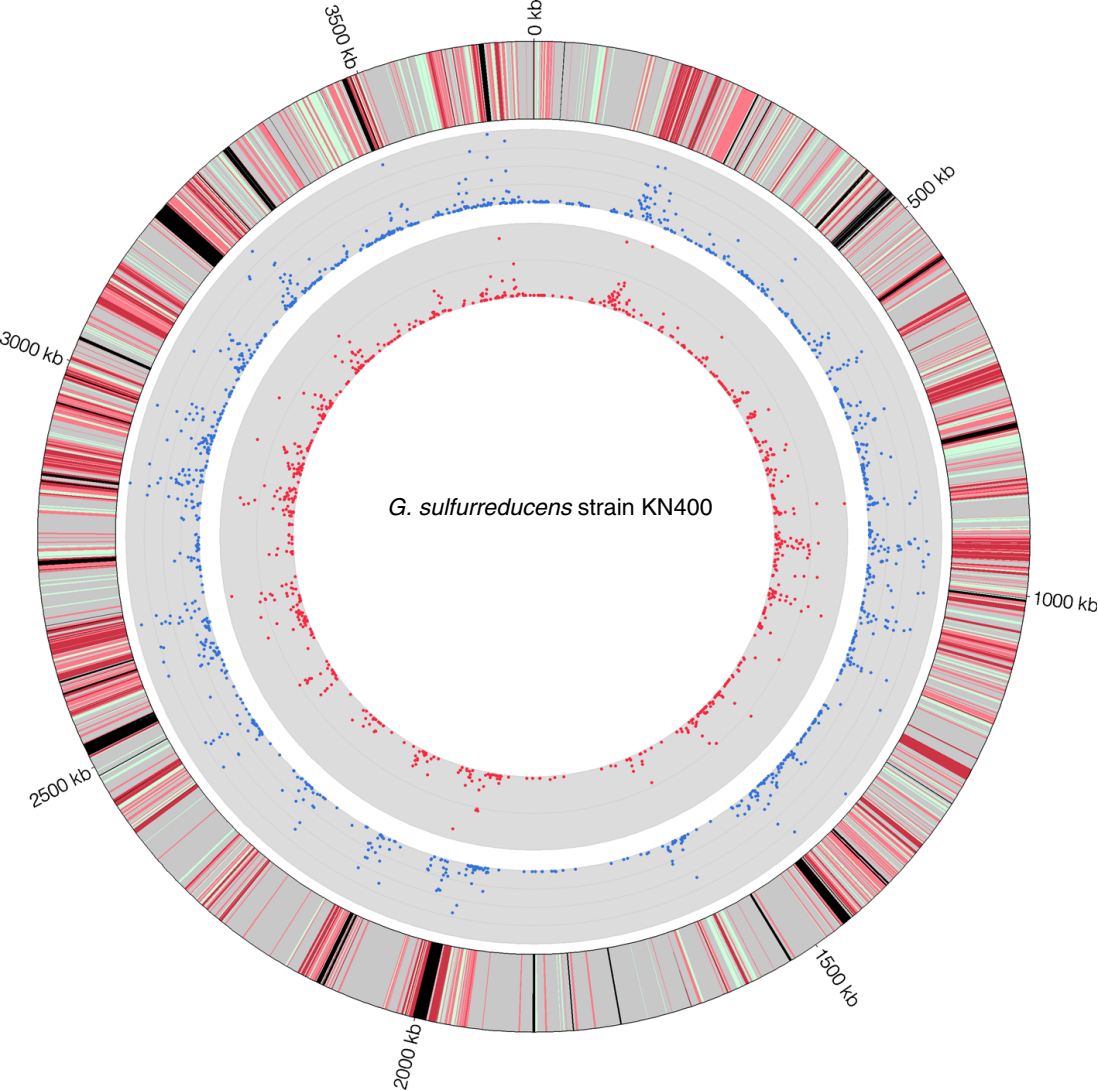
***G. sulfurreducens* strain KN400 genome differences compared to strain PCA.***Inner ring:* the number of non-synonymous (those that affect protein sequence) single nucleotide polymorphisms (SNP) per gene. Line scale = 25 SNP *Center ring:* the total number of SNP per gene. Line scale = 25 SNP. *Outer ring:* Gene-scale differences in orthologs. Genes with only silent mutations are green, those that have less than 12 non-synonymous SNP per kb (within two standard deviations of the mean) are light red, and those with at least 12 non-synonymous SNP are dark red. Identical genes are grey. Those genes that lack orthologs entirely are black

There were no orthologs in the PCA strain for 126 of the ORFs (3.8%) in the KN400 strain genome (Figure 1, Additional file 1: Table S3). In order to better to describe these genes and their evolutionary history, their locations and characteristics were mapped and their sequences were compared to proteins from the 10,291 organisms in the Reference Sequence database from the National Center for Biotechnology Information [9]. 

Seventeen of these 126 ORFs had no significant similarity to any gene in another organism, six were most similar to proteins in the PCA strain, and 37 had highest similarity to proteins in *Geobacteraceae* species other than PCA (Additional file 1: Table S3). The remaining ORFs were most similar to genes from a variety of phylogenetically diverse organisms including *Desulfovibrio*, *Syntrophobacter*, *Pseudomonas*, and *Vibrio* species (Additional file 1: Table S3), suggesting that there are diverse evolutionary histories for these genes.

The 126 genes are spread throughout the KN400 genome, the majority in clusters together (Additional file 2: Figure S1). In total, there are 18 regions in the KN400 genome where at least two consecutive genes have no ortholog in the PCA genome (Additional file 1: Table S4). Eight are bordered by or contain transposase or integrase genes, three are bordered by tRNA genes, and one is bordered by a conserved repeated nucleic acid sequence (Additional file 1: Table S4) – indicating that these regions may be or once were mobile genetic elements [10].

One strain-specific region, found at 466250–483475 in the KN400 genome (genes KN400_0440-KN400_0451), is inserted relative to PCA just downstream of a tRNA gene, between the orthologs to GSU0465 and GSU0466 (Additional file 1: Table S1). This region encodes a phage integrase and replisome organizer as well as a restriction/modification system. The two genes for the restriction/modification system (KN400_0449 and KN400_0451) are similar to genes from *Meiothermus ruber*, and are not found in any other *Geobacteraceae*, suggesting that this region may have been acquired by lateral gene transfer.

Another KN400 strain-specific region is found between 3479100–3485200. Here, KN400_3199-KN400_3206 are found between the orthologs to GSU3265 and GSU3267 (Additional file 1: Table S1). The genes include five conserved hypothetical proteins and three proteins involved in metal transport and usage: a metal-transporting ATPase, a metal-dependent phosphohydrolase, and a hybrid iron transport/cell signaling protein. All of these genes are most similar to those found in other *Geobacteraceae* and *Deltaproteobacteria* (Additional file 1: Table S1), suggesting that they might have been lost in the PCA strain genome.

In addition to regions of relative insertion in KN400, there are others where, between regions of excellent conservation between the two genomes, there is a stretch of sequence for which there is little or no similarity. For example, KN400_1871 and GSU1849 are orthologs, and KN400_1883 and GSU1857 are orthologs, but the region between them has low sequence similarity (Additional file 2: Figure S2, Additional file 1: Table S1). However, in both strains, the genes encode products involved in exopolysaccharide biosynthesis (Additional file 1: Table S1; Additional file 1: Table S2). This may be a region where mutation accumulation was relatively unconstrained, and these genes are attractive targets for studies of differences in the biofilms formed by strains PCA and KN400.

A few of the genes found in the KN400 genome but not the PCA genome are isolated, not in the 18 clusters described above. One of these that may have a substantial effect on metabolism is the catalase. The KN400_2748 gene encodes a KatE catalase [11], while the catalase of *G. sulfurreducens* strain PCA, GSU2100, encodes a KatG-type [11] of no sequence similarity. While each strain encodes only one of the two catalases, there is evidence that each once encoded the other. KN400 has a region with nucleic acid similarity to the *katG* gene (2278624–2280808 KN400), but a single-base deletion results in a frameshift halfway through it. In the PCA genome, the region 3089478–3089600 aligns with the 5’ end and the 3’ end of the *katE* gene in KN400, though 90% of the gene has been deleted. Both KatE and KatG detoxify reactive oxygen species, but their mechanism and expression are different [11]. Response to oxidative stress is especially important in Fe(III)-reducing, obligate anaerobes like *Geobacter sulfurreducens* – reactive oxygen species cause DNA damage and disable many respiratory enzymes, and the presence of ferrous iron can create more of these compounds [12]. A difference in response to oxidative stress between the strains could affect many aspects of growth.

### Genes unique to the PCA strain

249 ORFs in the PCA genome (7.2%) did not have orthologs in the KN400 genome (Additional file 1: Table S5). Gene ontology analysis showed that three types of genes were very highly enriched in this group relative to the whole genome: those involved in DNA transposition, DNA integration, and RNA-dependent DNA replication (Additional file 1: Table S6). As with strain KN400, these ORFs were mostly found in clusters within the PCA genome (Additional file 2: Figure S1). There are 18 regions in the PCA genome in which at least two consecutive genes have no orthologs in the KN400 genome, (Additional file 1: Table S7). Twelve of these are bordered by (or contain) transposases, three by a tRNA gene(s), and two by conserved nucleic acid sequences. Compared to the KN400 strain, there are more transposases and more diverse types of transposases associated with these regions.

Several of these non-orthologous regions in the PCA genome are much larger than any of those found in the KN400 genome (Additional file 2: Figure S1). In the KN400 genome, 4 of the non-orthologous regions have at least ten genes, and the largest has 22 genes. In the PCA genome, 10 regions have at least ten genes, and the largest has 89 genes (Additional file 1: Table S4 and Additional file 1: Table S7).

The largest region of non-orthology to KN400 is between 2316300–2393600 in the PCA genome (Additional file 1: Table S7). This region of 89 ORFs (GSU2105-GSU2183) is inserted relative to the KN400 genome between KN400_3465 and a tRNA gene at 2285100. Two-thirds of the proteins encoded in this region are proteins of unknown function or transposases. Other proteins encoded include five putative transcriptional regulators and two efflux pumps (Additional file 1: Table S5). The second largest region unique to the PCA genome is between 49500–81300 (Additional file 1: Table S7). This region of 26 ORFs (GSU3471-GSU0064) is inserted relative to strain KN400 between a tRNA gene and KN400_0040. The majority of the proteins encoded here are also transposases and proteins of unknown function. However, this region also encompasses the CRISPR1 locus, six CRISPR-associated genes and a toxin/antitoxin pair, genes involved in resistance to exogenous DNA and potentially in gene regulation [13,14].

In total, the KN400 genome is ca. 100 kb smaller than the PCA genome, with the difference due to the ca. 100 genes in KN400 with no ortholog in PCA and ca. 200 genes in PCA with no ortholog in KN400. These genes have a variety of different apparent histories – some are not found in any other organism, some are syntenic between the genomes but lack sequence homology, some appear to have been lost entirely in one of the strains, and some may have arisen from lateral gene transfer from unrelated species. Strain-specific genes of potential interest include a number for membrane transport and polysaccharide metabolism; a restriction/modification system; catalases; a disulfide bond formation operon unique to KN400; and a CRISPR locus unique to PCA. The evolutionary history of the mobile genetic elements that are enriched in these regions also warrants further investigation.

### Single nucleotide polymorphisms (SNP) between the PCA and KN400 genomes

Using an alignment of the two whole genomes, 27,270 single nucleotide polymorphism sites and small insertions or deletions between the two strains were identified (Figure 1, Additional file 1: Table S8). Of these, 23,773 (87%) were intragenic in the PCA strain, with a rate of 6.8 SNP/kb in coding regions and 10.5 SNP/kb in non-coding regions.

Within the 3192 genes that had orthologs in both genomes, there were 17,964 SNP and 182 small-scale insertions or deletions (Figure 1, Table 2). The large majority (72%) of the sequence differences between the KN400 and the PCA orthologs were silent, meaning that the SNP caused no changes to translation, and that the protein sequences of the orthologs were identical. The remaining 5109 SNP (28%) were non-synonymous – they caused a difference in amino acid sequence during translation (Figure 1, Table 2).

**Table 2:** Summary of the number and types of SNP in different classes of genes

**PCA**	**Total**	**KN400 orthologs**	**% with orthologs**	**Total SNP**	**Total NS SNP**	**% NS SNP**	**genes with SNP**	**% Genes w.SNP**	**Genes with NS SNP**	**% genes w.NS SNP**	**SNP/ gene**	**NS SNP/ gene**	**SNP/ SNPed gene**	**NS SNP/ SNPed gene**	**# genes ≥1.2% NS SNP**	**% genes ≥1.2% NS SNP**
**genes**	3432	3192	92.6	18146	5109	28.2	1160	36.3	786	24.6	5.6	1.6	15.5	4.4	114	3.6
**central metabolism**	85	85	100.0	180	37	20.6	34	40.0	17	20.0	2.1	0.4	5.3	1.1	1	1.2
**cytochromes**	95	94	98.9	1156	365	31.6	48	51.1	36	38.3	12.3	3.9	24.1	7.6	7	7.4
**increased expression**	93	85	91.4	1518	388	25.6	43	50.6	33	38.8	17.9	4.6	35.3	9.0	6	7.1

One-third of genes had a change of sequence between the strains − 1160 of the 3192 orthologs had at least one SNP, with an average of 5.6 per gene (15.5 per gene in genes that had at least one SNP) (Figure 1, Table 2). A rate of 34 SNP/kb was two standard deviations above average.

One-quarter of proteins had a change of sequence between the strains – 786 of the 3192 orthologs had at least one non-synonymous SNP, with an average of 1.6 per ortholog (4.4 per gene with at least one SNP) (Figure 1, Table 2). The set of genes that had at least one non-synonymous SNP was significantly enriched in genes for: *c*-type cytochromes, integral membrane proteins, phosphorus and phosphate metabolism, cofactor biosynthesis, and ATP-dependent enzymes with a wide variety of functions including: transporters and exporters, helicases, mismatch repair proteins, excinucleases, proteases, and many sensor histidine kinases (Additional file 1: Table S6).

Proteins were considered heavily mutated if the corresponding gene had at least 12 non-synonymous SNP/kb, which was two standard deviations above average (Table 2). 125 of the orthologs (3.6%) were heavily mutated (Table 3, Additional file 1: Table S9). The set of heavily mutated proteins was significantly enriched in *c*-type cytochromes and in membrane-bound transport proteins (Table 3, Additional file 1: Table S6).

**Table 3:** Orthologous genes in the KN400 and PCA strains that have the highest level of protein-changing single nucleotide polymorphisms per kb (proteins of unknown function not shown)

**Gene**	**Annotation**	**PCA ortholog**	**Non-synonymous SNP per 1000 bases**	**Total SNP**
KN400_0176	quinoline oxidoreductase, small subunit protein	GSU0200	17.0	25
KN400_0177	quinoline oxidoreductase, large subunit protein	GSU0201	21.6	174
KN400_0192	FMN-cofactor binding protein	GSU0217	12.4	20
KN400_0193	cytochrome c oxidase synthesis (SCO) factor	GSU0218	14.4	32
KN400_0196	cytochrome c oxidase, subunit IV	GSU0221	13.8	11
KN400_0197	cytochrome c oxidase, subunit II	GSU0222	12.0	44
KN400_0198	cytochrome c oxidase assembly factor	GSU0223	18.5	43
KN400_0202	DNA methyltransferase	GSU0227	19.6	53
KN400_0225	sensor histidine kinase	GSU0253	20.1	118
KN400_0281	DnaJ domain protein	GSU0313	15.6	21
KN400_0418	ribonuclease D	GSU0443	20.1	64
KN400_0545	DNA-3-methyladenine glycosylase I	GSU0567	19.1	28
KN400_0547	nicotinamidase-related cysteine hydrolase	GSU0569	21.9	29
KN400_0597	cytochrome c (OmcE)	GSU0618	25.8	44
KN400_0681	cytochrome c biogenesis, ResB	GSU0704	12.8	36
KN400_0803	sensor histidine kinase	GSU0822	13.7	68
KN400_0811	efflux pump, RND family, membrane fusion protein	GSU0829	40.2	116
KN400_0818	response regulator	GSU0837	12.2	8
KN400_0880	3-oxoalanine-generating family protein	GSU0897	17.1	55
KN400_0883	SAM-dependent methyltransferase	GSU0900	19.1	40
KN400_0885	response receiver	GSU3516	12.8	17
KN400_0887	sensor histidine kinase	GSU3518	16.8	68
KN400_0892	pyranopterin cofactor biosynthesis, MoeB	GSU0907	34.3	44
KN400_0893	pyranopterin cofactor biosynthesis, MoaD	GSU0908	32.0	22
KN400_0899	ATP-dependent RNA helicase RhlE	GSU0914	17.9	105
KN400_0908	ABC transporter, ATP-binding protein	GSU0922	12.4	75
KN400_0914	zinc-dependent peptidase, M16 family	GSU0928	16.0	77
KN400_0950	protease	GSU0969	16.3	73
KN400_0995	OmpA family outer membrane lipoprotein	GSU1013	15.0	37
KN400_0996	smr domain protein	GSU1014	26.4	40
KN400_1013	methyl-accepting chemotaxis sensory transducer	GSU3523	19.6	153
KN400_1014	methyl-accepting chemotaxis sensory transducer	GSU1035	15.2	107
KN400_1117	methyl-accepting chemotaxis sensory transducer	GSU1141	14.5	72
KN400_1307	cytochrome c	GSU1334	16.7	81
KN400_1319	sulfate ABC transporter, periplasmic sulfate-binding	GSU1346	20.7	58
KN400_1320	sulfate ABC transporter, membrane protein CysU	GSU1347	16.8	31
KN400_1510	transcriptional regulator, MarR family	GSU1483	49.0	59
KN400_1862	membrane-associated phosphatase, PAP2_like_5 family	GSU1840	12.2	15
KN400_1865	RNA exonuclease	GSU1843	18.2	58
KN400_1866	IPT/TIG domain protein	GSU1844	12.5	107
KN400_1884	IPT/TIG domain protein	GSU1858	25.1	173
KN400_2002	exopolysaccharide synthesis exosortase	GSU1979	12.6	38
KN400_2006	protein tyrosine kinase	GSU1983	14.0	27
KN400_2106	D-glycero-D-mannoheptose-1,7-bisphosphate phosphatase	GSU2084	12.2	20
KN400_2110	glycosyltransferase, group 2 family protein	GSU2088	57.0	139
KN400_2282	trehalose-6-phosphatase	GSU2336	26.6	41
KN400_2284	sodium/proton antiporter complex Mrp, protein G	GSU2338	43.8	52
KN400_2286	sodium/proton antiporter complex Mrp, protein E	GSU2340	40.2	62
KN400_2287	sodium/proton antiporter complex Mrp, protein D	GSU2341	16.5	106
KN400_2288	sodium/proton antiporter complex Mrp, protein C	GSU2342	46.6	18
KN400_2382	ATP-dependent protease, putative	GSU2433	12.2	77
KN400_2399	peptide methionine sulfoxide reductase	GSU2451	14.5	19
KN400_2420	TPR domain protein	GSU2476	17.6	74
KN400_2445	YVTN family beta-propeller domain protein	GSU3586	16.6	59
KN400_2449	cytochrome c (OmcS)	GSU2504	15.4	51
KN400_2452	sensor histidine kinase	GSU2507	13.9	70
KN400_2454	TPR domain protein	GSU2508	24.5	96
KN400_2457	sensor diguanylate cyclase/phosphoesterase	GSU2511	17.6	142
KN400_2460	cytochrome c	GSU2513	31.8	27
KN400_2624	2-dehydropantoate 2-reductase	GSU2683	38.5	104
KN400_2644	transcriptional regulator, TetR family	GSU2698	13.9	33
KN400_2649	molybdopterin-molybdenum ligase	GSU2703	12.3	59
KN400_2658	fibronectin type III domain protein	GSU2715	48.7	294
KN400_2660	hydrogenase, bidirectional NAD-reducing, protease	GSU2717	30.8	35
KN400_2668	cytochrome c	GSU2725	25.4	31
KN400_2716	transcriptional regulator, MerR family	GSU2779	18.0	30
KN400_2747	transcriptional regulator, Fur family	GSU2809	30.6	35
KN400_2750	glutaredoxin family protein	GSU2812	89.8	60
KN400_2997	NADPH ferredoxin oxidoreductase (FNOR) beta subunit	GSU3058	15.6	32
KN400_3000	squalene cyclase domain protein	GSU3061	12.3	107
KN400_3006	UDP-N-acetylenolpyruvylglucosamine reductase	GSU3067	21.1	58
KN400_3214	cytochrome c	GSU3274	28.8	32
KN400_3301	sensor histidine kinase	GSU3357	22.2	146
KN400_3348	OmpJ-related porin	GSU3403	29.9	97
KN400_3368	dihydrolipoamide dehydrogenase-related protein	GSU3424	16.4	86

Some genes had only silent SNP, or sequence changes that did not affect the resulting protein sequence, indicating that changes to their sequence may be under negative selective pressure. Solely silent changes were present in 373 of the orthologs (12%) (Additional file 1: Table S1). This set of genes was significantly enriched in genes involved in flagellum biogenesis and amino acid biosynthesis (Additional file 1: Table S6). The KN400 strain, unlike the PCA strain, produces flagella and is motile [5]. The most notable silent-mutation-rich region of the genome was cluster GSU1095-GSU1102, which encodes a phosphate transporter and phosphate-dependent regulatory genes (Additional file 1: Table S2).

In order to better understand the phenotypic effects of the nucleotide polymorphisms between the strains, several metabolic networks involved in electron transfer out of the cell were examined in more detail.

### Sequence differences in central energy metabolism

*G. sulfurreducens* primarily grows by coupling the oxidation of acetate to the reduction of extracellular electron acceptors. Acetate is oxidized by the TCA cycle, with the products used to generate a proton gradient for ATP synthesis [1,15]. The electrons that remain from this process are transferred out of the cell to Fe(III) or to electrodes.

In total, 65 genes in strain PCA encode the proteins involved in acetate oxidation, and the KN400 strain has orthologs to each of these (Additional file 1: Table S10). Ten of the 65 contain a protein sequence change, with fewer per gene than the genome as a whole (0.4 *versus* 1.6 non-synonymous SNP per gene) (Table 2). None are highly mutated.

Separately from energy generation, a portion of acetate is shunted to a different pathway and used as the sole source for biomass synthesis via the gluconeogenic and fatty acid pathways [15,16]. 20 genes are involved in this other fate of acetate and the KN400 strain has orthologs to each of these (Additional file 1: Table S10). However, two reactions from this pathway show higher sequence changes between the strains – phosphate acetyltransferase and acetate kinase (PAT - GSU2706, AK - GSU2707). In KN400 the acetyltransferase has 38 SNP (8 non-synonymous) and the kinase has 45 SNP (7 non-synonymous), with a 27-fold higher non-synonymous SNP/kb ratio than the other enzymes of central energy metabolism (Table 2, Additional file 1: Table S10).

The sequence differences in PAT and AK stand out as the most pronounced in the otherwise very well-conserved enzymes of central energy metabolism. It has previously been shown that strain PCA cannot grow on acetate alone if either PAT or AK is knocked out [16]. This phenotype arises because the CoA-transferase that activates acetate for oxidation in the TCA cycle requires CoA derived from the TCA cycle itself. Therefore, additional pathways are needed to produce acetyl-CoA for biosynthetic pathways. With acetate as the sole growth substrate, this anaplerotic function is performed by AK and PAT.

Differences in these two enzymes could have a substantial effect on the flux between acetate oxidation and reduction, and thereby on electron transport rates out of the cell and on biomass production. A lower flux through AK/PAT relative to the TCA cycle would translate to a decreased biomass yield per molecule of acetate oxidized and a higher ratio of electrons transferred to the electrode per cell – the phenotype seen in the KN400 strain [5].

### Sequence differences in extra-cytoplasmic electron transfer proteins

*G. sulfurreducens* grows by using Fe(III) oxide or energy-harvesting electrodes as the terminal electron acceptor [2,17]. Cytochromes, pili, and exopolysaccharides have all been shown to be important for growth using these extracellular electron acceptors [1].

For both the PCA and KN400 strains, the whole proteomes were scanned for the heme-binding motif of *c*-type cytochromes [18] (Additional file 1: Table S11). In each strain, 135 genes encode proteins with this C-X-X-C-H motif (Additional file 1: Table S11). Since this definition of cytochrome is minimal, proteins were excluded as cytochromes if they had a predicted function or cellular localization that indicated that they were not involved in electron transport outside the cell; most were proteins with iron-sulfur-binding domains (Additional file 1: Table S11).

In total, 107 genes in the PCA and 106 in the KN400 strain were predicted to encode *c*-type cytochromes (Additional file 1: Table S11). Only one of the PCA cytochromes lacked an ortholog in KN400: GSU2515, a monoheme cytochrome that has not been shown to be involved in electron transport. Fifty-four of the 106 *c*-type cytochromes contain at least one SNP in KN400 relative to PCA (51%), compared to 36% of genes in the whole genome (Table 2).

There were more than two-fold more SNP per cytochrome compared to the genome as a whole: 12.3 vs 5.6 SNP/gene, and almost twice as many of the encoded proteins were heavily mutated: 7.4% vs 3.6% (Table 2). However, all 106 of the orthologous cytochromes had the same number of predicted heme-binding sites, with up to 27 motifs in a single protein (Additional file 1: Table S11). So, while the cytochromes have more than the average number of changes to nucleotide and protein sequences, these changes do not result in a change to the number of hemes predicted to be bound to each cytochrome. This indicates that the proteins’ electron carrying capacity may still function, though perhaps with a changed redox potential due to structural differences [19].

Most of the cytochromes previously shown to be required for growth with Fe(III) or for optimal current production [1] are identical in KN400 and PCA, including PpcA, MacA, OmcB, OmcC, OmcF, and OmcZ (Additional file 1: Table S11).

However, two cytochromes are among the most heavily mutated genes in the genomes and are required for extracellular electron transfer – OmcE and OmcS (GSU0618, GSU2504) (Table 3, Additional file 1: Table S11). The *omcS* gene is found in a region (2743500–2775500 in PCA) that has one of the highest SNP rates in the genome: 36 SNP/kb. Of the 22 genes encoded, 8 are heavily mutated, one is frameshifted, and three lack orthologs in the KN400 strain (Additional file 1: Table S2). This region also encodes six other *c*-type cytochromes: GSU2494, GSU2495, GSU2501, GSU2503 (*omcT*), GSU2513 and GSU2515. Finding significant changes to OmcS in particular is especially relevant in the KN400 strain because this cytochrome has been shown to be localized outside of the cell along the length of the pili [20] where it may facilitate electron transfer from pili to the terminal electron acceptor [6,21]. Changes to the electron-carrying protein at this key position for electrical contact could have a substantial effect on phenotype.

Pili are also required for the reduction of extracellular electron acceptors by *G. sulfurreducens*[21,22]. They are believed to provide a conduit for electron transfer through biofilms and to the cytochromes responsible for Fe(III) oxide reduction [6]. There are 24 genes involved in biogenesis and assembly of pili (GSU0146, GSU0230, GSU0436, GSU1063-GSU1066, GSU1491-GSU1496, GSU2028-GSU2038, GSU3548, GSU2043). All 24 genes have orthologs in the KN400 strain (Additional file 1: Table S1), and they are all well conserved – 20 of the genes are identical at the nucleotide level between the strains, and only one has a single non-synonymous mutation, a PilT homolog (GSU0436).

Extracellular polysaccharides are also required for electrode reduction; they are involved in anchoring the *c*-type cytochromes that provide the electrical conduit between the cell and the electrode surface [23]. Five of the genes in the extracellular anchoring polysaccharide gene cluster (GSU1498 to GSU1508) have non-synonymous SNP (Additional file 1: Table S2), including one in the an ABC transporter ATPase subunit required for polysaccharide production [23].

Finally, OmpB and OmpC are putative copper-binding oxidoreductases required for insoluble Fe(III) reduction in *G. sulfurreducens*[24,25]. The *ompB* gene (GSU1394) is identical between the two strains, and *ompC* (GSU2657) has a single synonymous mutation.

Thus between the KN400 and the PCA strains there is both conservation and divergence among the extra-cytoplasmic proteins involved in transferring electrons out of the cell. Most previously known to be required *in vivo* did not have large sequence differences, including six cytochromes, two copper proteins, and the pilin. However, cytochromes considered as a class were much more likely to contain non-synonymous SNP, and two important cytochromes, OmcS and OmcE, have especially large differences. Notably though, not a single heme-binding site was lost due to the changes. These data support previous analyses showing that a diversity of cytochromes are characteristic of *Geobacter* species, but individual cytochromes tend to be poorly conserved [26]. This kind of cytochrome diversity and adaptability may be especially important for the use of terminal electron acceptors with a wide variety of redox potentials.

### Sequence differences in the genes with increased expression during growth on an electrode

Two previous studies have analyzed changes in gene expression in the PCA strain during growth on an energy-harvesting electrode. Given the improvements in electron transfer to electrodes in the KN400 strain relative to PCA, any sequence changes in these genes are of particular interest. The first study looked at gene expression in the early stages of anode biofilm formation, and showed that 93 genes had an increase in transcript abundance compared to growth by Fe(III) reduction [27]. The second looked at expression changes in established, high-current-density biofilms, and showed that 13 genes had an increase in transcript abundance compared to growth with fumarate as an electron acceptor [22].

Only one of the 13 genes with increased expression on the high-current electrode had substantial changes between the KN400 and the PCA strain. OmpJ is an outer-membrane channel protein known to influence the quantity and localization of cytochromes in *G. sulfurreducens*[28], so changes to its sequence could influence electron transfer very broadly. OmpJ (GSU3403) has 3-fold higher expression during growth on the electrode, and it was among the most highly mutated genes between the two strains, with 30 non-synonymous changes per kb (Additional file 1: Table S9).

Twenty-four genes have increased expression during early-stage growth on the electrode and also had either no ortholog or large sequence differences in the KN400 strain (Table 4). The *omcS* cytochrome gene is the most highly up-regulated of all genes and it was among the proteins with the largest sequence difference between the KN400 and PCA strains (discussed above). A third copper-binding oxidoreductase has increased expression during growth on the electrode [27], and the cluster encoding this multicopper oxidase, as well as a cytochrome *d* and two copper chaperone proteins (GSU1251-GSU1257) had large sequence differences (Additional file 1: Table S2). Unlike OmpB and OmpC (mentioned above), the role of these proteins in electron transfer has not been studied, though interestingly the most similar complex is found in the Fe(II)-oxidizing *Leptothrix* species [29]. 

**Table 4:** Genes in strain PCA that have increased expression during growth with an electrode as the electron acceptor and that lack orthologs or have a high level of protein-changing single nucleotide polymorphisms per kb

**PCA gene**	**Annotation**	**KN400 ortholog**	**Non-synonymous SNPs per 1000 bases**	**electrode increase expression (fold-change)**
GSU0062	TraD protein	none		1.34
GSU0618	cytochrome c (OmcE)	KN400_0597	25.8	1.98
GSU0829	efflux pump, membrane fusion protein	KN400_0811	40.2	1.69
GSU0955	RNA-directed DNA polymerase	none		1.76
GSU1253	cytochrome d	KN400_1227	13.7	1.65
GSU1844	IPT/TIG domain protein	KN400_1866	12.5	1.55
GSU2113	transcriptional regulator	none		1.4
GSU2129	conserved hypothetical protein	none		1.38
GSU2133	lipoprotein	none		1.62
GSU2135	metal efflux pump, inner membrane protein	none		1.73
GSU2136	metal efflux pump, membrane fusion protein	none		1.88
GSU2137	metal efflux pump, outer membrane protein	none		1.94
GSU2139	transposase	none		1.28
GSU2143	conserved hypothetical protein	none		3.82
GSU2471	RNA-directed DNA polymerase	none		1.56
GSU2476	TPR domain protein	KN400_2420	17.6	1.49
GSU2497	lipoprotein	KN400_2442	23.8	1.32
GSU2504	cytochrome c (OmcS)	KN400_2449	15.4	19.46
GSU2507	sensor histidine kinase	KN400_2452	13.9	1.41
GSU2508	TPR domain protein	KN400_2454	24.5	1.37
GSU2690	VacJ family lipoprotein	KN400_2637	36.6	1.35
GSU2773	conserved domain protein	none		1.62
GSU2779	transcriptional regulator	KN400_2716	18.0	3.77
GSU3067	UDP-N-acetylenolpyruvylglucosamine reductase	KN400_3006	21.1	1.32
GSU3403*	OmpJ porin	KN400_3348	29.9	2.76

In addition to these copper proteins, a transcriptional regulator (GSU2779) with homology to CueR, a copper-responsive transcriptional activator [30], has a four-fold increase in expression on the electrode and was heavily mutated in the KN400 strain (Table 4). The CueR protein is broadly involved in metal homeostasis, and three general metal efflux pumps also have increased expression on the electrode and sequence changes in KN400. The first pump (GSU2135-GSU2137) was one of the few proteins in PCA that lacked orthologs in KN400 (Table 4), and two others have non-synonymous SNPs (GSU0829-GSU0830 and GSU1338-GSU1341).

Immediately downstream of one of the metal pumps, a fourth important transporter has large differences between the KN400 and PCA strains: the only sulfate transporter in the genome (GSU1350-GSU1352) was heavily mutated in KN400 (Table 3). This is upstream of three additional genes for sulfur metabolism: a sulfite reductase, a sulfur carrier protein, and a thiocarboxylate synthase (GSU1350-GSU1352), which are among the few in PCA that lack orthologs entirely in KN400 (Additional file 1: Table S5). These changes indicate that KN400 may have a significant difference in its usage of sulfur, copper, and perhaps other metals, which could have far-reaching impact on the types of redox proteins active in the KN400 strain.

## Conclusions

The KN400 strain of *G. sulfurreducens* was isolated from a fuel cell that was run for several months poised at increasingly lower potentials. Its 16S rRNA genes are identical to those in the well-studied PCA strain, but the quantity of nucleotide polymorphisms and their strong enrichment at silent sites, as well as the diversity and distribution of genes that lack orthologs between the genomes all suggest the strains have been subject to substantial selective pressure since their divergence. Further study of strain variation in *G. sulfurreducens* – these two are the only strains that have been sequenced – and the analysis of more closely related genomes would help to reconstruct a detailed evolutionary history, particularly of the genomic mobility seen in this species.

The KN400 strain has a number of favorable characteristics that make it the preferred strain to grow in a microbial fuel cell. The first is speed of electron transfer: KN400 oxidizes the donor and transfers the resulting electrons to the electrode faster than PCA [5]. Analysis of the differences between the two genomes suggests (and eliminates) several explanations for this phenotype. KN400 does not contain additional genes that PCA lacks which are predicted to be involved in donor oxidation, nor does it contain any large sequence changes to the enzymes for acetate oxidation via the TCA cycle. However, there are significant changes to two anaplerotic enzymes that could shift the carbon flux in the KN400 strain from biomass production towards more rapid oxidation of acetyl-CoA. If these two enzymes have lower activity in KN400, the TCA cycle may complete full turns more often, and this would increase the fraction of acetate that is oxidized for respiration.

Interestingly, the increased respiration rate does not lead to increased cell growth in the KN400 strain [5], indicating that there may be important changes in ATP synthesis or availability. Previously, a PCA strain engineered with an unproductive ATP sink exhibited a similar phenotype [31]. In the KN400 genome, a very broad range of ATP-dependent enzymes were more likely to have sequence changes, and the phosphate sensor, transporter and several phosphate regulators were all also changed. Thus, the phosphate levels, the ATP:ADP ratio, and the energy state of the cell may be different in the KN400 strain relative to the PCA strain, which could result in broad metabolic differences.

The second favorable characteristic of KN400 when grown in a microbial fuel cell is its highly conductive biofilms [6] from which low-potential electrons can be harvested. Analysis of the differences between the two genomes also suggests (and eliminates) factors that may contribute to this phenotype. Though pili are the conductive material in KN400 biofilms, there are no differences in the pilin subunit, biogenesis genes, nor the PilR transcriptional regulator between the strains. However the cell-electrode connection is also mediated by *c*-type cytochromes, and there are substantial differences between the strains in these proteins. The sequences of both cytochromes and of the channel proteins that affect their localization have substantial changes, most notably in the OmcS cytochrome that is physically associated with the pili. In addition, there are changes to several clusters of exopolysaccharide biosynthesis genes in KN400 which may further affect cytochrome localization and general conductivity. Excellent conservation of heme-binding sites despite sequence changes indicates that cytochrome redox potential changes also warrant further investigation in the KN400 strain. Finally, sensing and response to global redox potential and metal homeostasis may have significant differences in KN400 due to changes in proteins involved in the transport and metabolism of copper, of sulfur, and of metals generally. These types of changes would have a broad impact on electron transport in the species.

## Methods

### Genome reannotation

In regions of nucleotide sequence conservation where an ORF was predicted in only one of the genomes, ORFs were added if the gene products had predicted functions; if they were conserved in genomes other than *G. sulfurreducens*; if they had multiple homologs in *G. sulfurreducens*; or if they had plausible predicted ribosome-binding sites. ORFs were removed if they overlapped other features such as ORFs, tRNAs, CRISPRs (clustered regularly interspaced short palindromic repeats), riboswitches, or strong hairpins. In total 162 ORFs or pseudogenes were added to the genome of strain PCA, and 158 ORFs or pseudogenes were removed (from the original 3469 ORFs and pseudogenes). In total 108 ORFs or pseudogenes were added to the genome of strain KN400 and 177 were removed (from the original 3421 ORFs and pseudogenes) (Additional file 1: Table S1 and Additional file 1: Table S2).

Discrepancies in start coordinates between homologous genes in strain KN400 and strain PCA were resolved by identifying plausible ribosome-binding sites. In cases where no homologs were detected in any other species and both predicted starts possessed ribosome-binding sites, the protein sequences were examined for the presence of a short N-terminal hydrophobic segment indicating a putative signal peptide. Start sites that placed signal peptides nearer to the N-terminus were preferred. In the absence of a signal peptide, the start site resulting in a longer ORF was preferred. Finally, in a few cases the discrepancies in start sites were due to a frameshift that was present in the ortholog in one of the strains but absent in the other (Additional file 1: Table S1 and Additional file 1: Table S2). Stop coordinates were adjusted only if there were predicted selenocysteine codons, frameshifts, or nonsense codons interrupting an alignment of protein sequences. In total 403 start sites were changed in the strain PCA genome, and 435 start sites were changed in the strain KN400 genome. Six stop sites were changed in the strain PCA genome, and 16 stop sites were changed in the strain KN400 genome.

To determine functional annotation and most closely related species, all ORFs in both genomes were compared to the National Center for Biotechnology Information Reference Sequence Database [9], the Conserved Domain Database [32], MicrobesOnline comparative genomics databases [33], and the *G. sulfurreducens* metabolic model [15].

### Ortholog prediction

The PCA and KN400 genome sequences were aligned using progressive Mauve version 2.3.0 with a match seed weight of 15 and the HOXD scoring matrix [34] and manual refinement, and orthologs were predicted based on this alignment. Orthologs were also identified without considering genome position by identifying reciprocal best BLAST matches in all-against-all proteome comparisons [35]. Differences in these methods were resolved as described in the text. Genome figures were made using Circos [36] and Mauve.

### Polymorphism detection

Custom scripts (Additional file 3) were written to identify differences between the sequences of the strains, including single nucleotide polymorphisms and insertions and deletions, and to calculate protein percent identity, using the Mauve alignment as the input. Sequence differences in the coding regions were determined to have synonymous or non-synonymous effects using the standard bacterial genetic code. Where there was more than one sequence change in the triplet, each was considered independently.

### Functional enrichment

Gene ontology functional enrichment was done using DAVID software [37]. The background gene set was all genes in the *G. sulfurreducens* PCA genome. All test sets had at least 100 genes. Enrichment was tested for the functional categories derived from the gene ontology database [38], the COG database [39], the Protein Information Resource database [40], and the InterPro database [41]. Enrichment in function in the test set of genes versus the whole-genome background was determined with a modified Fisher’s exact test (EASE score) [37], with p-value cut-offs at either 0.05 or 0.01 for significance.

## Competing interests

The authors declare that they have no competing interests.

## Authors’ contributions

JB designed the comparative experiments, analyzed the polymorphism data and wrote the manuscript; MA re-annotated the genomes and helped write the manuscript; NY wrote the analysis scripts and helped with the comparative analysis; DL helped write the manuscript. All authors read and approved the final manuscript.

## Supplementary Material

Additional file 1 **Table S1.** – KN400 genome; **Table S2.** – PCA genome; **Table S3.** – KN400 genes that lack an ortholog in the PCA strain; **Table S4.** – regions in the KN400 genome that lack an ortholog in the PCA strain; **Table S5.** – PCA genes that lack an ortholog in the KN400 strain; **Table S6.** – Gene ontology enrichment analysis; **Table S7.** – Regions in the PCA genome that lack an ortholog in the KN400 strain; **Table S8.** – Single nucleotide polymorphisms across the genome; **Table S9.** – Heavily mutated proteins; **Table S10.** – Polymorphisms in the genes of central metabolism; **Table S11.** – Polymorphisms in the cytochromes. Click here for file

Additional file 2 **Figure S1.** Alignment of the genomes of *G. sulfurreducens* strain PCA and strain KN400. Figure S2. A large region of low similarity between the KN400 and PCA genomes. Click here for file

Additional file 3 Scripts for polymorphism detection. Click here for file
