# Supplementary material for: Comparative genomic analysis of Geobacter sulfurreducens KN400, a strain with enhanced capacity for extracellular electron transfer and electricity production
Source: BMC Genomics. 2012 Sep 12;13:471. doi: 10.1186/1471-2164-13-471 (PMC3495685; doi:10.1186/1471-2164-13-471)
Supplement: Additional file 2 — Figure S1. Alignment of the genomes of G. sulfurreducens strain PCA and strain KN400. Figure S2. A large region of low similarity between the KN400 and PCA genomes. [file 1471-2164-13-471-S2.pdf]

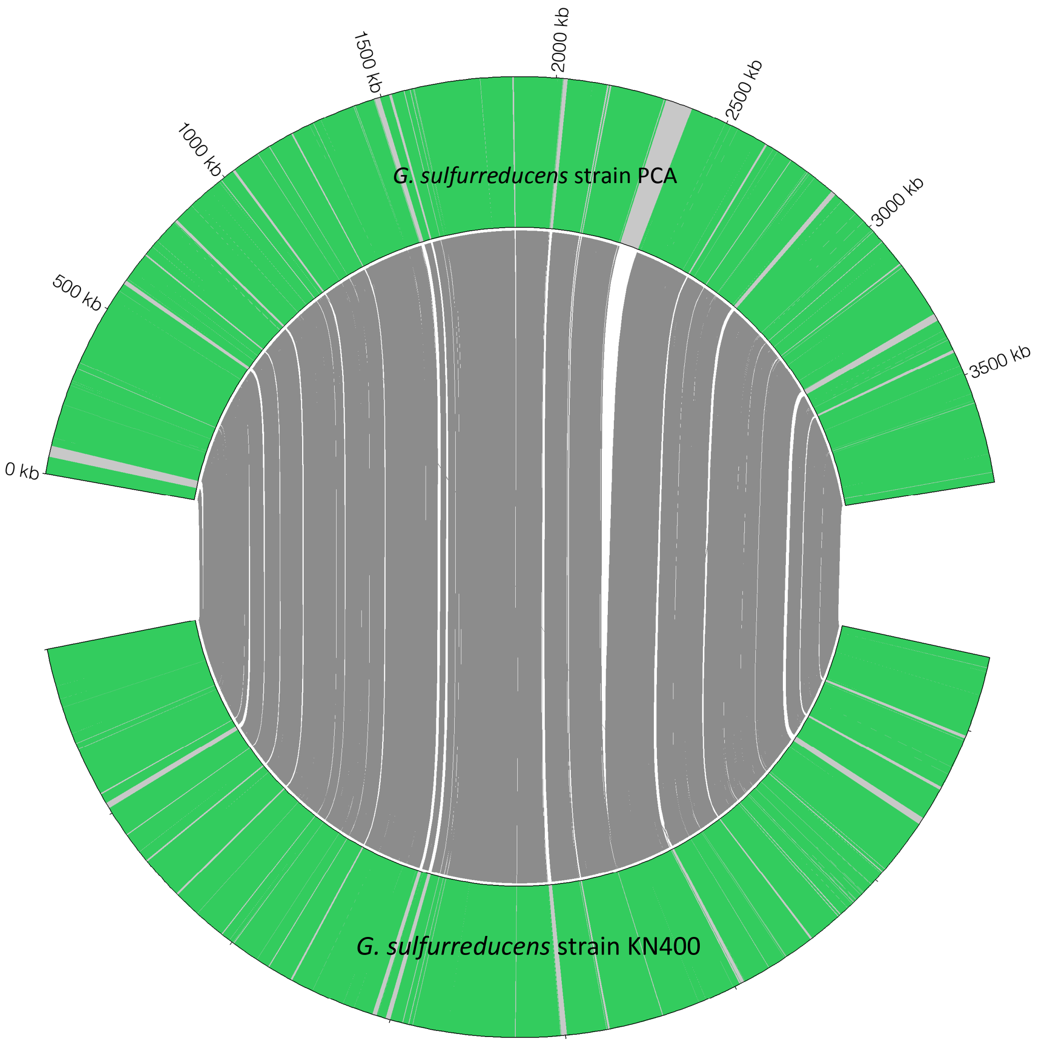


**Supplementary Figure 1.**

**Alignment of the genomes of *G. sulfurreducens* strain PCA and strain KN400.**

Positional mapping of *G. sulfurreducens* strain PCA to *G. sulfurreducens* strain KN400 based on the whole-genome nucleotide alignment. Orthologs are shown in green and connected with lines between the genomes. Regions that lack orthologs (are specific to the strain) are shown in grey and lack connections.

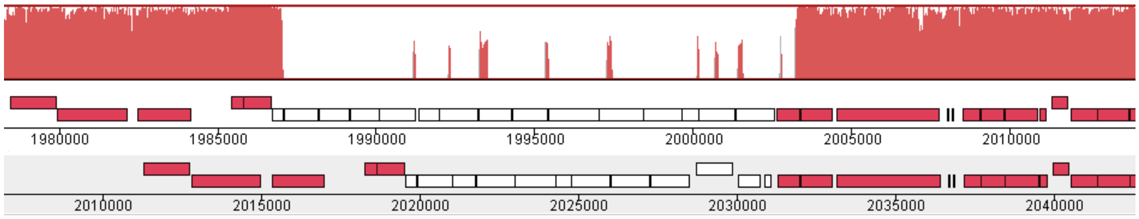


**Supplementary Figure 2.**
**A large region of low similarity between the KN400 and PCA genomes.**
The upper panel shows the nucleotide sequence identity between the two strains in red, and the lower panel shows the open reading frames in the region in each genome. Orthologous genes are red and those that lack orthologs are white.
