# Supplementary material for: Comparative genomic analysis of Geobacter sulfurreducens KN400, a strain with enhanced capacity for extracellular electron transfer and electricity production
Source: BMC Genomics. 2012 Sep 12;13:471. doi: 10.1186/1471-2164-13-471 (PMC3495685; doi:10.1186/1471-2164-13-471)
Supplement: Additional file 3 — Scripts for polymorphism detection. [file 1471-2164-13-471-S3.pdf]

mutations in coding vs noncoding regions

```
#!/usr/bin/env ruby -w


require 'bio'

require 'getoptlong'

require 'rubygems'


include Bio


# == Synopsis

#

# Calculates the frequency of snps in a progMauve .snps file, comparing those SNPs within a

# specified set of ranges (coding regions) to the rest of snps in the file. Coding regions are taken

# from a progMauve .orthologs file.

#

# == Usage

# ./cod_v_non.rb -s gs2-KN400_p2.trsnps -o gs2-KN400_p2.orthologs

#

# -s is followed by a Mauve-generated SNP list.

#

# -o is followed by a pMauve .orthologs file containing ranges in the following format:

# 0:GSU0000.1:30-13671:KN400_0001:30-1367

#

#

# == Author

# Ned Young, Lovley Lab, U Mass Microbiology

# 8/21/09
```

```
# based on bias.rb, 8/20/09

# based on support_snps.rb, 7/13/09

# based on compare_snps.rb, 4/1/09

# based on twelvers.rb, 12/05/08

# based on reseq_grep, 8/07/08

# based on load_orves.rb, 2/07/08

# based on annotaid.rb, 1/29/08

# based on ortho_table.rb, 1/28/08

# based on assghi.rb, 1/8/08

# based on core.pl, 5/21/07

#


##################################################################################
##################


######################

# METHOD DEFINITIONS #

######################


# =get_ranges_2_ways

#

# Gets ranges from a file

# Gets passed a filename

# Returns 3 arrays of ranges ( start..end )


def get_ranges_2_ways( r_fn )

	r_fh = File.new( r_fn )

	all_ranges = Array.new
```

```
	paired_only = Array.new
	mut_strain_ranges = Array.new
	r_fh.each do |line|
		raw = line.chomp.split(/:/)  # colon defines field limits
		if raw[4] =~ /(\d+)-(\d+)/ then
			first = $1.to_i
			last = $2.to_i
			mut_strain_ranges.push( first..last )
		end
		if  raw[2] =~ /(\d+)-(\d+)\s+1/ then  # paired
			first = $1.to_i
			last = $2.to_i
			paired_only.push( first..last )
			all_ranges.push( first..last )
		elsif ( ( raw[1] =~ /Gsul/ ) and ( raw[2] =~ /(\d+)-(\d+)/ ) ) then  # not paired
			first = $1.to_i
			last = $2.to_i
			all_ranges.push( first..last )
		end
	end
	return all_ranges, paired_only, mut_strain_ranges
end

# =get_m_muts_pro
#
# Makes array of SNP positions from the Mauve-generated SNP list.
# Gets passed a fh for the SNP list.
```

```
# Returns array of arrays, last SNP.

def get_m_muts_pro( mln_rpt_fh )
	m_muts = Array.new
	current_one = 0
	keys = %w( change gen1_pos gen2_pos )
	mln_rpt_fh.each do |line|
		next if line =~ /SNP/  # Skips first line
		line_stuff = grab_data_from_report( line, keys )
		current_one = line_stuff[ 'gen1_pos' ].to_i
		mut_change = line_stuff[ 'change' ]
		mut_genome_pos = line_stuff[ 'gen2_pos' ].to_i
		mut = [ current_one, mut_change, mut_genome_pos ]
		m_muts.push( mut )
	end
	return m_muts, current_one
end

# =grab_data_from_report
#
# A method to take a line of tab-delimited data fields,
# read them into a hash with the field names as keys, and return the hash
# Gets passed a line of data in a string, and the field names (keys)
# Returns the hash

def grab_data_from_report( line, keys )
	raw = line.chomp.split(/\t/)  # tab defines field limits
```

```
        raw.shift if raw[ 0 ] == ""
        line_stuff = Hash.new
        counter = 0
        raw.each do |element|
                line_stuff[ keys[ counter] ] = element
                counter +=1
        end
        return line_stuff
end

# =in_mut_genome_orf
#
# Looks for a particular position in a set of ranges
# Gets passed a pos and an array of ranges
# Returns true (in ranges) or false

def in_mut_genome_orf( pos, ranges )
        is_in = false
        mut_range_found_in = nil
        ranges.each do |range|
                in_range = range === pos # Checks to see if pos is in range
                if in_range == true then
                        is_in = true
                        mut_range_found_in = range
                        next
                end
        end
```

```
    return is_in, mut_range_found_in
end

##########################################################################################
##################

# Main program

# First check that arg(s) are given on the command line
fail "\nUsage: #$0 -s gs2-KN400_p2.trsnps -o gs2-KN400_p2.orthologs\n" if ARGV.size < 2

opts = GetoptLong.new(
    [ "-s",                 GetoptLong::REQUIRED_ARGUMENT ],
    [ "-o",                 GetoptLong::REQUIRED_ARGUMENT ]
)
s_rpt_fn, orthos_fn = nil
opts.each do |opt, arg|
    case opt
        when '-s'
            s_rpt_fn = arg
        when '-o'
            orthos_fn = arg
    end
end

# get the ranges (1st genome)
all_ranges, paired_only, mut_strain_ranges = get_ranges_2_ways( orthos_fn )
```

```
# This part makes array of SNPs from the snps list (e.g. mauve_parse output). At the moment, it needs

# to be in the same folder as this program.  Uses first genome position.

s_rpt_fh = File.new( s_rpt_fn )

m_muts, last_snp = get_m_muts_pro( s_rpt_fh )


puts "m_muts: #{m_muts.size}"

                                        #temp


# Sum ranges from all_ranges

all_ranges_sum = 0

all_ranges.each do |range|

	r_size = ( ( range.last ) - ( range.first ) ) + 1

	all_ranges_sum = all_ranges_sum + r_size

end

# Calculate amount not in ranges

not_in_ar = last_snp - all_ranges_sum


# Sum ranges from paired_only

paired_only_sum = 0

paired_only.each do |range|

	r_size = ( ( range.last ) - ( range.first ) ) + 1

	paired_only_sum = paired_only_sum + r_size

end

# Calculate amount not in ranges

not_in_po = last_snp - paired_only_sum
```

```
# Prepare report fh and print report
nameparts = s_rpt_fn.split(/\./)
nameparts.pop
bias_fn = nameparts.push("bias_report").join("\.")
report = File.new( bias_fn, "w" )

in_ar_count, out_ar_count = 0, 0
in_po_count, out_po_count = 0, 0
in_bp_count, out_bp_count = 0, 0
snps_in_orfs = Array.new
snps_in_orf_pairs = Array.new
snps_in_broad_pairs = Array.new
m_muts.each do |mut|
	snp, change, mut_genome_pos = mut

	is_in_po = false
	paired_only.each do |range|
		in_range = range === snp # Checks to see if snp is in range
		if in_range then
			is_in_po = true
			next
		end
	end
	# Identifies SNPs in paired orthologs
	if is_in_po == true then
		in_po_count += 1
		snps_in_orf_pairs.push( mut )
```

```
            in_bp_count += 1
            snps_in_broad_pairs.push( mut )
        else
            out_po_count += 1
            out_bp_count += 1  # Tentative.  Might be subtracted back later.
        end

        is_in_orf = false
        found_in = nil
        all_ranges.each do |range|
            in_range = range === snp # Checks to see if snp is in range
            if in_range == true then
                is_in_orf = true
                found_in = range
                next
            end
        end
        # Identifies SNPs in any 1st genome coding region
        if is_in_orf  then
            in_ar_count += 1
            snps_in_orfs.push( mut )
            # Even if not in an ortholog pair, the SNP might still align to protein in the 2nd
genome.
            if is_in_po == false
                in_mut_orf, mut_range_found_in = in_mut_genome_orf( mut_genome_pos,
mut_strain_ranges )
                if in_mut_orf
                    in_bp_count += 1
```

```
                    snps_in_broad_pairs.push( mut )

                    out_bp_count -= 1  # Subtract back.

                    puts "SNP #{snp} is found in GSU range #{found_in}, and also is at
#{mut_genome_pos} in KN400; found in #{mut_range_found_in}."
                        #temp

                end

            end

        else

            out_ar_count += 1

        end

end


report.puts "SNPs in protein on both genomes:"

snps_in_orf_pairs.each {|s| report.puts s.join("\t")}


# Calculate frequency of snp occurence (all_ranges)

in_ar_freq = in_ar_count.to_f / all_ranges_sum.to_f

out_ar_freq = out_ar_count.to_f / not_in_ar.to_f


# Calculate frequency of snp occurence (paired_only)

in_po_freq = in_po_count.to_f / paired_only_sum.to_f

out_po_freq = out_po_count.to_f / not_in_po.to_f


report.puts "Amount of sequence within \"all_ranges\":          #{all_ranges_sum}"

report.puts "Amount of sequence outside of \"all_ranges\":      #{not_in_ar}\n"

report.puts "Number of SNP occurrence within \"all_ranges\":    #{in_ar_count}"

report.puts "Number of SNP occurrence outside of \"all_ranges\": #{out_ar_count}"

report.puts "Frequency of SNP occurrence within \"all_ranges\":    #{in_ar_freq}"
```

```
report.puts "Frequency of SNP occurrence outside of \"all_ranges\": #{out_ar_freq}"
report.puts "Ratio (in_ar_freq/out_ar_freq):                              #{in_ar_freq/out_ar_freq}\n\n"

report.puts "Number of SNP occurrence within \"broad_pairings\":     #{in_bp_count}"
report.puts "Number of SNP occurrence outside of \"broad_pairings\": #{out_bp_count}"

report.puts "Amount of sequence within \"paired_orthologs\":          #{paired_only_sum}"
report.puts "Amount of sequence outside of \"paired_orthologs\":       #{not_in_po}\n"
report.puts "Number of SNP occurrence within \"paired_orthologs\":     #{in_po_count}"
report.puts "Number of SNP occurrence outside of \"paired_orthologs\": #{out_po_count}"
report.puts "Frequency of SNP occurrence within \"paired_orthologs\":     #{in_po_freq}"
report.puts "Frequency of SNP occurrence outside of \"paired_orthologs\": #{out_po_freq}"
report.puts "Ratio (in_po_freq/out_po_freq):                              #{in_po_freq/out_po_freq}\n\n"

puts "Amount of sequence within \"all_ranges\":          #{all_ranges_sum}"
puts "Amount of sequence outside of \"all_ranges\":       #{not_in_ar}\n"
puts "Number of SNP occurrence within \"all_ranges\":     #{in_ar_count}"
puts "Number of SNP occurrence outside of \"all_ranges\": #{out_ar_count}"
puts "Frequency of SNP occurrence within \"all_ranges\":     #{in_ar_freq}"
puts "Frequency of SNP occurrence outside of \"all_ranges\": #{out_ar_freq}"
puts "Ratio (in_ar_freq/out_ar_freq):                              #{in_ar_freq/out_ar_freq}\n\n"

puts "Number of SNP occurrence within \"broad_pairings\":     #{in_bp_count}"
puts "Number of SNP occurrence outside of \"broad_pairings\": #{out_bp_count}\n"

puts "Amount of sequence within \"paired_orthologs\":          #{paired_only_sum}"
puts "Amount of sequence outside of \"paired_orthologs\":       #{not_in_po}\n"
```

```
puts "Number of SNP occurrence within \"paired_orthologs\":     #{in_po_count}"

puts "Number of SNP occurrence outside of \"paired_orthologs\": #{out_po_count}"

puts "Frequency of SNP occurrence within \"paired_orthologs\":     #{in_po_freq}"

puts "Frequency of SNP occurrence outside of \"paired_orthologs\": #{out_po_freq}"

puts "Ratio (in_po_freq/out_po_freq):                              #{in_po_freq/out_po_freq}\n\n"


exit
```

------

mutations synonymous or nonsynoymous

```
#!/usr/bin/env ruby -w


require 'bio'

require 'getoptlong'

require 'rubygems'


include Bio


# == Synopsis

#

# Calculates the frequency of synonymous and nonsyn. SNPs in a progMauve .orthologs.alignments file.

#

# == Usage

# ./syn_v_non.rb -o gs2-KN400_p2.orthologs.alignments -x gs2.gbk -y KN400.gbk

#

# -o is followed by a pMauve .orthologs.alignments file.

#
```

```
# -x and -y are each followed by the filename for a .gbk file

#

# Note: uses position #s from the first genome (Genome "0").

#

# == Author

# Ned Young, Lovley Lab, U Mass Microbiology

# 8/21/09

# based on cod_v_non.rb, 8/21/09

# based on bias.rb, 8/20/09

# based on support_snps.rb, 7/13/09

# based on compare_snps.rb, 4/1/09

# based on twelvers.rb, 12/05/08

# based on reseq_grep, 8/07/08

# based on load_orves.rb, 2/07/08

# based on annotaid.rb, 1/29/08

# based on ortho_table.rb, 1/28/08

# based on assghi.rb, 1/8/08

# based on core.pl, 5/21/07

#


##################################################################################
##################


######################

# METHOD DEFINITIONS #

######################


# =process_ortho_pair
```

```
#

# Takes a single pair of seqs and looks for differences.

# Gets passed a pair of records, 2 output fhs, and 3 counters.  Also two proteome hashes, so that

# the proteins can be looked up.  Also the filename of the second genome (e.g. KN400.gbk).

# Returns 3 counters, updated, and a string giving info about stop differences, if any.


def process_ortho_pair( records, report, table, s_count, n_count, i_count, gen1_orfs, gen2_orfs,
gen2_fn )

	mut_set = Array.new

	top_seq, bot_seq, top_start, top_stop, bot_start, bot_stop, top_gene, bot_gene, strand = nil

	records.each do |record|

		next if record == "\n"

		portions = record.split(/\n/)

		header = portions[ 0 ]

		portions.shift

		seq = portions.join

		if header =~ /(\d):\d+\-\d+:([\w\.]+)\s([-\+])/ then

			genome = $1

			gene =   $2

			strand = $3

		else

			puts "Header not readable: #{header}"

		end

		if genome == "0" then

			top_seq = Bio::Sequence::NA.new( seq )

			top_gene = gene

		else

			bot_seq = Bio::Sequence::NA.new( seq )
```

```
			bot_gene = gene
		end
	end
	report.print "#{top_gene}=#{bot_gene}:  "
	table.print "#{top_gene}\t#{bot_gene}\t"

	# Flip if on opposite strand
	if strand == "-" then
		top_seq = top_seq.reverse_complement
		bot_seq = bot_seq.reverse_complement
	end

	# Address length dif. by padding end, if necessary-- probably only affects last pair
	should_skip = false
	if top_seq.length < bot_seq.length then
		dif = bot_seq.length - top_seq.length
		tail = ''
		for i in 1..dif
			tail = tail + "-"
		end
		top_seq = top_seq + tail
		puts "Lengthened a top seq w/ gaps to match bot seq."
		should_skip = true
	end
	if bot_seq.length < top_seq.length then
		dif = top_seq.length - bot_seq.length
		tail = ''
```

```
		for i in 1..dif
			tail = tail + "-"
		end
		bot_seq = bot_seq + tail
		puts "Lengthened a bot seq w/ gaps to match top seq."
		should_skip = true
	end

	# See if the two (original, from .gbk file) ORFs are the same length
	stop_diff = ''
	if should_skip == false then
		# Get orfs from the orf hashes
		if !gen1_orfs[ top_gene ] then
			puts "Couldn't find info for #{top_gene} in genome 1."
			report.puts "Couldn't find info for #{top_gene} in genome 1."
			table.puts "Couldn't find info for #{top_gene} in genome 1."
			return s_count, n_count, i_count, stop_diff
		end
		top_orf_info = gen1_orfs[ top_gene ]
		top_orf_seq, top_orf_start_pos, top_orf_end_pos, top_orient = top_orf_info
		#puts "top_gene: #{top_gene}, end pos: #{top_orf_end_pos}"
														#temp
		if !gen2_orfs[ bot_gene ] then
			puts "Couldn't find info for #{bot_gene} in genome 2."
			report.puts "Couldn't find info for #{bot_gene} in genome 2."
			table.puts "Couldn't find info for #{bot_gene} in genome 2."
			return s_count, n_count, i_count, stop_diff
```

```
            end
            bot_orf_info = gen2_orfs[ bot_gene ]
            bot_orf_seq, bot_orf_start_pos, bot_orf_end_pos, bot_orient = bot_orf_info
            top_start = top_orf_start_pos
            top_stop = top_orf_end_pos
            bot_start = bot_orf_start_pos
            bot_stop = bot_orf_end_pos
            top_orf_len = top_orf_end_pos - top_orf_start_pos + 1     # Getting these from the
.gbk is best.
            bot_orf_len = bot_orf_end_pos - bot_orf_start_pos + 1
            if top_orf_len != bot_orf_len then
                top_seq, bot_seq, stop_diff = recalc_pair( top_seq.upcase, bot_seq.upcase,
top_orf_seq.upcase,
                    bot_orf_seq.upcase, gen2_fn, top_orient )
            end

            # Go through each base pair of the alignment.
            ali_len = top_seq.length
            top_gap_len, bot_gap_len = nil
            top_codon = String.new
            bot_codon = String.new
            top_trails = 0
            bot_trails = 0
            positions_three = Array.new
            for i in 0..( ali_len - 1 )
                if strand == "-" then
                    pos = top_stop - i  # In case of - strand, abs. position counts
backwards from the end.
                else
```

```
            pos = top_start + i
        end
        top_base = top_seq[ i, 1 ]
        bot_base = bot_seq[ i, 1 ]
        next unless top_base =~ /[acgtACGT\-]/
        next unless bot_base =~ /[acgtACGT\-]/



        if top_base == "-" then
            mut_set, i_count, top_gap_len, bot_gap_len = check_for_indel(
"top", pos, strand, top_gap_len, bot_gap_len, mut_set, i_count )
            if top_gap_len then
                top_gap_len += 1
            else
                top_gap_len = 1
            end
            top_codon = String.new
            bot_codon = String.new
        elsif bot_base == "-" then
            mut_set, i_count, top_gap_len, bot_gap_len = check_for_indel(
"bot", pos, strand, top_gap_len, bot_gap_len, mut_set, i_count )
            if bot_gap_len then
                bot_gap_len += 1
            else
                bot_gap_len = 1
            end
            top_codon = String.new
```

```
                                        bot_codon = String.new
                                else   # Both seqs have a base
                                        mut_set, i_count, top_gap_len, bot_gap_len = check_for_indel(
"none", pos, strand, top_gap_len, bot_gap_len, mut_set, i_count )
                                        # Add bases to a growing codon pair
                                        top_codon = top_codon + top_base
                                        bot_codon = bot_codon + bot_base
                                        if ( ( top_codon.length >= 3 ) and ( ( i + 1 ).remainder( 3 ) == 0 ) ) then
                                                if top_codon.length > 3 then
                                                        top_codon = top_codon[ -3, 3 ]   # The last 3 bases
                                                        bot_codon = bot_codon[ -3, 3 ]   # The last 3 bases
                                                        # Process the initial 1 or 2 bases separately
                                                        if strand == "-" then
                                                                pos_to_pass = pos + ( top_codon.length - 1 )
# First base of the codon
                                                        else
                                                                pos_to_pass = pos - ( top_codon.length - 1 )
# First base of the codon
                                                        end
                                                        len_minus_3 = top_codon.length - 3
                                                        top_bases = top_codon[ 0, len_minus_3 ]   # The first
base(s)
                                                        bot_bases = bot_codon[ 0, len_minus_3 ]   # The first
base(s)
                                                        mut_set, s_count, n_count = check_bases(
top_bases, bot_bases, pos_to_pass, mut_set, s_count, n_count )

                                                end
                                                # Process the codon
                                                if strand == "-" then
```

```
                        pos_to_pass = pos + 2  # First base of the codon
                    else
                        pos_to_pass = pos - 2  # First base of the codon
                    end
                    mut_set, s_count, n_count = check_codon( top_codon,
bot_codon, pos_to_pass, strand, mut_set, s_count, n_count )
                    top_codon = String.new
                    bot_codon = String.new
                end
            end
        end
    end

    # Finish writing line to the report
    mut_set.push( stop_diff )
    report.puts mut_set.join(' | ')
    return s_count, n_count, i_count, stop_diff
end

# =check_for_indel
#
# Checks to see if an indel mutation has just been finished (the last base is the current base).
# Gets passed a code indicating gap, if any, in the current character, as well as the positon, the
# strand, the running totals of recent gap characters, the mutations array and the indel count.
# Return updated the mutations array, indel count and running totals, which have been reset as
# appropriate.
```

```
def check_for_indel( code, pos, strand, top_gap_len, bot_gap_len, mut_set, i_count )

        if ( ( ( code == "top" ) or ( code == "none" ) ) and bot_gap_len ) then   # Bottom genome gap
is over.

                indel_len = bot_gap_len

                indel_pos = pos - 1

                mut_set.push( "I#{indel_len}:#{indel_pos}" )

                i_count += 1

                bot_gap_len = nil

        elsif ( ( ( code == "bot" ) or ( code == "none" ) ) and top_gap_len )   # Top genome gap is over.

                indel_len = top_gap_len

                if strand == "-" then

                        indel_start = pos + 1

                        indel_end = pos + ( indel_len )

                else

                        indel_start = pos - ( indel_len )

                        indel_end = pos - 1

                end

                mut_set.push( "D#{indel_len}:#{indel_start}-#{indel_end}" )

                i_count += 1

                top_gap_len = nil

        end

        return mut_set, i_count, top_gap_len, bot_gap_len

end


# =check_codon

#

# Checks a pair of 3 bp sequences for mutation and if so, whether syn. or nonsyn.

# Gets passed the two 3bp seqs as strings, the absolute position of the 1st base, the strand, the
```

```
# mutations string for the gene, the syn. and nonsyn. counts for the genome.
# Returns updated mutations string for the gene as well as updated syn. and nonsyn. counts for the
# genome.

def check_codon( top_codon, bot_codon, pos, strand, mut_set, s_count, n_count )
	unless top_codon == bot_codon
		top_codon = Bio::Sequence::NA.new( top_codon )
		bot_codon = Bio::Sequence::NA.new( bot_codon )
		is_it_nonsyn = !(top_codon.translate == bot_codon.translate)
		n_called = false
		for i in 0..2 do
			if strand == "-" then
				current_pos = pos - i
			else
				current_pos = pos + i
			end
			top_base = top_codon[i]
			bot_base = bot_codon[i]
			unless top_base == bot_base
				if is_it_nonsyn
					# If there is more than one mutation in this codon, they need
not both be called "N"
					how_many = count_how_many( top_codon, bot_codon )
					if how_many > 1 then
						dif_solo = check_if_solo( top_base, i, bot_codon )
						if dif_solo then
							mut_set.push(
"N#{current_pos}:#{top_base.upcase}-#{bot_base.upcase}" )
```

```
                        n_count += 1
                        n_called = true
                    else
                        # Should usually be a "S" mut.  However, if it
is in the 3rd position and no "N" has
                        # been called for this codon yet, then it has
to be "N".
                        if ( ( i == 2 ) and ( n_called == false ) ) then
                            mut_set.push(
"N#{current_pos}:#{top_base.upcase}-#{bot_base.upcase}" )
                            n_count += 1
                        else
                            mut_set.push(
"S#{current_pos}:#{top_base.upcase}-#{bot_base.upcase}" )
                            s_count += 1
                        end
                    end
                else
                    mut_set.push(
"N#{current_pos}:#{top_base.upcase}-#{bot_base.upcase}" )
                    n_count += 1
                end
            else
                mut_set.push( "S#{current_pos}:#{top_base.upcase}-
#{bot_base.upcase}" )
                s_count += 1
            end
        end
    end
end
```

```
        return mut_set, s_count, n_count
end


# =check_bases
#
# Checks a pair of 1 bp ( or 2 bp) sequences for mutation and if so, considers them syn.
# Gets passed the two seqs as strings, the absolute position of the 1st base, the mutations
# string for the gene, the syn. and nonsyn. counts for the genome.
# Returns updated mutations string for the gene as well as updated syn. and nonsyn. counts for the
# genome.


def check_bases( top_codon, bot_codon, pos, mut_set, s_count, n_count )
        unless top_codon == bot_codon
                top_codon = Bio::Sequence::NA.new( top_codon )
                bot_codon = Bio::Sequence::NA.new( bot_codon )
                seq_len = top_codon.length
                for i in 1..seq_len do
                        current_pos = ( pos + i ) - 1
                        top_base = top_codon[i]
                        bot_base = bot_codon[i]
                        unless top_base == bot_base
                                mut_set.push( "S#{current_pos}:#{top_base.upcase}-
#{bot_base.upcase}" )
                                s_count += 1
                        end
                end
        end
        return mut_set, s_count, n_count
```

```
end

# =count_how_many
#
# Counts the number of differing positions.
# Gets passed two Bio::Sequence::NA objects, each made from a 3bp string.
# Returns number differing.

def count_how_many( top_codon, bot_codon )
	count = 0
	for i in 1..3 do
		count += 1 unless top_codon[i] == bot_codon[i]
	end
	return count
end

# =check_if_solo
#
# Checks if mutation would result in a different aa.
# Gets passed a base, codon_pos and a Bio::Sequence::NA object.
# Returns true if mutation would result in a different aa, else false.

def check_if_solo( top_base, codon_pos, bot_codon )
	top_codon = bot_codon.dup
	top_codon[codon_pos, 1] = top_base  # Creates a copy of bot_codon w/just one difference
	dif_solo = !(top_codon.translate == bot_codon.translate)
end
```

```
# =recalc_pair

#

# Finds out whether there are leading or trailing gp characters when the two ORFs are aligned.

# Gets passed the two seqs from the Mauve output, which need trimming, as well as the two seqs
from

# the ORFs.  Also the filename of the second genome (e.g. KN400.gbk).

# Returns trimmed sequences, as well as an array of info for a line in the output table on

# start/stop site differences.


def recalc_pair( top_seq, bot_seq, top_orf_seq, bot_orf_seq, gen2_fn, top_orient )


        nameparts = gen2_fn.split(/\./)

        nameparts.pop

        gen2_fn = nameparts.join("\.")

        top_lead, top_trail, top_orf_lead, top_orf_trail = needleroozer( top_seq, top_orf_seq )

        msg = ''

        if [ top_lead, top_trail, top_orf_lead, top_orf_trail ].max > 0 then

                # Top seq differs from it's ORF.  Report difference.


                if top_orf_lead > 0

                        msg = 'Start is ' + top_orf_lead.to_s + ' bp earlier in ' + gen2_fn + '. '

                        # Trim the sequence start to match the GSU ORF.

                        start_advance = top_orf_lead

                        top_seq = top_seq.slice(start_advance..-1)

                        # Replace the bot seq with one that has been trimmed to match top seq.

                        bot_seq = bot_seq.slice(start_advance..-1)

                end
```

```
        if top_orf_trail > 0
            msg = msg + 'Stop is ' + top_orf_trail.to_s + ' bp later in ' + gen2_fn + '.'
            # Trim the sequence stop to match the GSU ORF.
            stop_shorten = top_orf_trail
            top_seq = top_seq.slice(0..-top_orf_trail)
            # Replace the bot seq with one that has been trimmed to match top seq.
            bot_seq = bot_seq.slice(0..-top_orf_trail)
        end
    else  # Bottom seq differs from it's ORF.  Don't replace.  Report difference.
        bot_lead, bot_trail, bot_orf_lead, bot_orf_trail = needleroozer( bot_seq,
bot_orf_seq )
        if bot_orf_lead > 0
            msg = msg + 'Start is ' + bot_orf_lead.to_s + ' bp later in ' + gen2_fn + '. '
        end
        if bot_orf_trail > 0
            msg = msg + 'Stop is ' + bot_orf_trail.to_s + ' bp earlier in ' + gen2_fn + '.'
        end
    end
    #puts msg

                                                    #temp
    return top_seq, bot_seq, msg
end


# =get_orfs_hash
#
# Makes a hash of untranslated, protein-coding ORFs from a genome file.
# incoming argument is a filename for the genome's NCBI genome file (.gbk)
```

```
# returns the hash

def get_orfs_hash( gen_fn )
	# File needs to be in the same directory as this program
	fh = File.new( gen_fn, "r" )
	proteins = Hash.new
	ff = Bio::FlatFile.new(Bio::GenBank, fh)
	# iterates over each GenBank entry
	ff.each_entry do |gb|
		# iterates over each element in 'features'
		gb.features.each do |feature|
			position = feature.position
			attribs = feature.assoc		# put into Hash
			# skips the entry if "/translation=" is not found; restricts following to protein
seqs.
	next unless attribs['translation']
			locus = attribs['locus_tag']
			if position =~ /complement\((\d+)\.\.(\d+)/ then
				end_pos = $1.to_i
				start_pos = $2.to_i
				strand = '-'
			elsif  position =~ /(\d+)\.\.(\d+)/
				start_pos = $1.to_i
				end_pos = $2.to_i
				strand = '+'
			end
			seq =  gb.naseq.splicing(position)  # nucleic acid sequence
			proteins[ locus ] =  [ seq, start_pos, end_pos, strand ]
```

```
        end
    end
    return proteins
end


# =needleroozer
#
# Does a needle alignment, counts leading or trailing gap characters, reports these in terms of
# start/stop site shifted =/- x bp in genome 2.  Also edits 2nd sequence to have same begin and end
# as the first.
# Gets passed two seqs.
# Returns info on difs, edited sequence.


def needleroozer( x_sequence, y_sequence )
    # Put them in temp files
    seq1_fh = File.new( "seq1.tmp", "w" )
    seq1_fh.puts ">x_sequence\n#{x_sequence}"
    seq2_fh = File.new( "seq2.tmp", "w" )
    seq2_fh.puts ">y_sequence\n#{y_sequence}"
    seq1_fh.close
    seq2_fh.close


    f = IO.popen("needle seq1.tmp seq2.tmp -gapopen 10 -gapextend 0.5 -outfile stdout")
    top_seq, bot_seq = '', ''
    finished = false
    f.each( "\nx_sequence" ) do |record| # Chops file into records separated by "x_sequence"
        break if finished == true
```

```
        portions = record.split(/\n/)
        if record =~ /^##/      # The first record
            next
        end
        finished = true if portions.size > 6  # Last portion has more lines
        if portions[0] =~ /(^\s+(\d+)\s+)([\w\-]+)/
            top_seq = top_seq + $3
        else
            puts "Top seq not correctly parsed."
        end
        if portions[2] =~ /(^[\w\.]+\s+(\d+)\s+)([\w\-]+)/
            #leader_len = $1.length
            #start_pos = $2.to_i
            bot_seq = bot_seq + $3
        else
            puts "Bottom seq not correctly parsed."
        end
    end
    f.close
    File.unlink("seq1.tmp")
    File.unlink("seq2.tmp")
    # Count leading and trailing gap characters, if any.
    top_lead, top_trail = lead_trail( top_seq )
    bot_lead, bot_trail = lead_trail( bot_seq )
    return top_lead, top_trail, bot_lead, bot_trail
end
```

```
# =lead_trail
#
# Counts leading and trailing gap characters.
# Gets passed a string containing the sequence.
# Returns number of leading, trailing gap characters, respectively.

def lead_trail( seq )
	whole_lead = nil
	lead_is_past = false
	lead = 0
	trail = 0
	for i in 0..(seq.size - 1) do
		if seq[i] == "-"
			lead += 1 if lead
			trail += 1
		else
			whole_lead = lead unless lead_is_past
			lead_is_past = true
			lead = nil
			trail = 0
		end
		whole_lead = seq.size unless lead_is_past
	end
	return whole_lead, trail
end

##################################################################################
##################
```

```
# Main program

# First check that arg(s) are given on the command line
fail "\nUsage: #$0 -o gs2-KN400_p2.orthologs.alignments -x gs2.gbk -y KN400.gbk\n" if ARGV.size < 3

opts = GetoptLong.new(
	[ "-o",				GetoptLong::REQUIRED_ARGUMENT ],
	[ "-x",				GetoptLong::REQUIRED_ARGUMENT ],
	[ "-y",				GetoptLong::REQUIRED_ARGUMENT ]
)
orthos_fn, gen1_fn, gen2_fn = nil
opts.each do |opt, arg|
	case opt
		when '-o'
			orthos_fn = arg
		when '-x'
			gen1_fn = arg
		when '-y'
			gen2_fn = arg
	end
end

gen1_orfs = get_orfs_hash( gen1_fn )
gen2_orfs = get_orfs_hash( gen2_fn )

# Prepare report fh
```

```
nameparts = orthos_fn.split(/\./)

nameparts.pop

nameparts.pop

s_n_fn = nameparts.push("s_n_report").join("\.")

report = File.new( s_n_fn, "w" )


# Prepare table fh

nameparts.pop

table_fn = nameparts.push("s_n.tdt").join("\.")

table = File.new( table_fn, "w" )


report.puts "Mutations found.\nSource: #{orthos_fn}\n#{Time.now}\nNote: When an insertion position is given, it indicates an insertion immediately after that position.\n\n"

table.puts "G.s gene\tKN400 gene\tSNPs\tnonsyn.\tindels\tstop change"

s_count, n_count, i_count = 0, 0, 0

old_s, old_n, old_i = 0, 0, 0


# Loop thru the ortholog pairs in the file

IO.foreach(orthos_fn, "=") do |pair|

        next if pair == "="

        pair.slice!(-1)

        records = pair.split(/>/)

        records.shift if records[0] == ""

        s_count, n_count, i_count, stop_diff = process_ortho_pair( records, report, table, s_count, n_count, i_count, gen1_orfs, gen2_orfs, gen2_fn )

        s_inc = s_count - old_s

        n_inc = n_count - old_n

        i_inc = i_count - old_i
```

```ruby
        table.print "#{s_inc + n_inc}\t#{n_inc}\t#{i_inc}\t#{stop_diff}\n"
        old_s, old_n, old_i = s_count, n_count, i_count
end

report.puts "\nSynonymous mutations in ORFs: #{s_count}"
report.puts "Non-synonymous mutations in ORFs: #{n_count}"
report.puts "Indel mutations in ORFs: #{i_count}"

exit
```
